# Tremor Reduction at the Palm of a Parkinson’s Patient Using Dynamic Vibration Absorber

**DOI:** 10.3390/bioengineering3030018

**Published:** 2016-07-05

**Authors:** Sarah Gebai, Mohammad Hammoud, Ali Hallal, Hassan Khachfe

**Affiliations:** 1 Department of Mechanical Engineering, Lebanese International University, Beirut, Lebanon; sarah.gebai@gmail.com (S.G.); ali.hallal@liu.edu.lb (A.H.); 2 Department of Biomedical Engineering, Lebanese International University, Beirut, Lebanon; hassan.khachfe@liu.edu.lb

**Keywords:** Parkinson’s disease, involuntary tremor, tuned vibration absorber, dynamically coupled system

## Abstract

Parkinson’s patients suffer from severe tremor due to an abnormality in their central oscillator. Medications used to decrease involuntary antagonistic muscles contraction can threaten their life. However, mechanical vibration absorbers can be used as an alternative treatment. The objective of this study is to provide a dynamic modeling of the human hand that describes the biodynamic response of Parkinson’s patients and to design an effective tuned vibration absorber able to suppress their pathological tremor. The hand is modeled as a three degrees-of-freedom (DOF) system describing the flexion motion at the proximal joints on the horizontal plane. Resting tremor is modeled as dual harmonic excitation due to shoulder and elbow muscle activation operating at resonance frequencies. The performance of the single dynamic vibration absorber (DVA) is studied when attached to the forearm and compared with the dual DVA tuned at both excitation frequencies. Equations of motion are derived and solved using the complex transfer function of the non-Lagrangian system. The absorber’s systems are designed as a stainless steel alloy cantilevered beam with an attached copper mass. The dual DVA was the most efficient absorber which reduces 98.3%–99.5%, 97.0%–97.3% and 97.4%–97.5% of the Parkinson’s tremor amplitude at the shoulder, elbow and wrist joint.

## 1. Introduction

Tremor is considered as the most common abnormal involuntary movement disorder and the source of functional disability [1]. The involuntary tremor of healthy people is called ‘physiological tremor’ and both Parkinson’s disease (PD) and essential tremor are considered as ‘pathological tremors’. Movement disorders cause patients with pathological tremors to have significant uncontrollable hand tremor movement [2].

PD is a multi-system neurodegenerative disorder caused by a lack in the level of dopamine (>80% of dopamine in the brain). PD tremor is related to resting and postural tremor. Rest tremor is the most recognizable sign of PD, and it initiates usually in upper limbs. Rest and posture tremor are in the range of 3–7 Hz and 5–12 Hz [3], respectively. Essential tremor is a bilateral of kinetic and posture tremor in the range of 4–12 Hz [4]. The negative correlation between amplitude and frequency justifies the significant movement in hand of PD patients when the person is at resting conditions.

Tremor in hand of PD patients makes them suffer while performing their daily tasks and they can feel embarrassed to face other people. Pharmacological treatments can be applied to different types of tremor. Levodopa drug and propranolol can be used to reduce the resting tremor, primidone and propranolol for hand postural tremor, beta-blockers for kinetic tremor and primidone and anticholinergic medication for essential tremor. Medication can decrease tremor progress, but it has withdrawal and side effects like addiction [5]. In case of non-responsive or medication failure, brain stereotactic surgery can be applied. These treatments are like lesioning surgery, Gamma-Knife radiosurgery, and deep brain stimulation (DBS). DBS may reduce tremor, but it has a direct impact on the influences of neuronal activity patterns in the basal ganglia loops. Medication and neurosurgical procedures can have diverse effects like: ataxia, confusion, muscle paralysis, hallucinations, speech impediment, stroke, hemiparesis and brain hemorrhage. In addition, positive effects of those treatments are temporary and 25% of patients can lose their life quality since they do not respond to drugs or neurosurgery treatments [6,7]. Each method has its weakness and may have high risks involving brain operation which points to the need for an alternative approach to reduce the vibration. As a result, mechanical treatment may be used as a good solution to suppress the tremor instead of using medical and surgical methods.

To reduce tremor caused by problems with supplying certain commands to muscles, a vibration absorber can be placed on the muscles to counteract the vibration. The dynamic vibration absorber (DVA) is a passive vibration controller added as a secondary system to reduce the steady state vibrational motion of the structure at a particular frequency. Absorbers’ parameters are chosen to minimize vibration at an undesired frequency. Oscillations can be translated into the movements of masses and springs due to the nature of the complex joint-muscle-tendon system [8]. Hashemi et al. [9] proposed a two degree-of-freedom (DOF) biodynamic model of upper limb with mass concentrated at centroid and inertia. Hand was modeled on the horizontal plane as two rigid segments to describe the flexion-extension planar motion of elbow and shoulder joints with sinusoidal excitation, but motion at wrist joint was not included. He designed a one DOF passive tuned vibration absorber modeled as a pendulum that was able to suppress rest tremor in the elbow and shoulder of a PD patient. However, the human hand is excited by muscular activity over a range of driving frequencies which may confirm the need for a multi-vibration absorber. Rahnavard et al. [10] used the same hand model and designed a single DOF absorber using the $H_{2}$ optimization method.

Igusa [11] studied the multiple mass dampers tuned within a frequency range. He found that it is more effective than tuned mass damper with the same total mass. Brennan [12] demonstrated the used of parallel multiple vibration absorbers tuned at slightly different frequencies which results in an improved broadband device.

In this paper, the human hand is modeled on the horizontal plane as a three DOF system by considering the flexion motion of the palm. Realistic parameters of the hand are used to size the hand segments and reflect its behavior. The mass of the upper arm, forearm and the palm are considered to be concentrated at its real centroid. However, Hashemi et al. [9] used a two DOF model with hand segments considered as uniform rods with masses concentrated at the centroid. He assumes that the forearm and the palm are the same rod. This model gives no information about the motion transmitted to the palm and the wrist joint. The single joint shoulder and elbow muscles are only considered to produce movement. The model’s sinusoidal input moment was due to elbow muscle activation which is operating at a single frequency neighborhood. In our model, the movements are considered due to shoulder, elbow and wrist single joint muscles and the Biceps a double joint muscle. The active input moments are due to the shoulder and elbow muscles’ operation at the first two resonance frequencies. However, these models do not take into consideration the pronation-supination motion at the elbow joint and the radial-ulnar deviations at the wrist joint which give more details on the real behavior of the hand in order to correctly address the involuntary tremor problems.

Based on his model, Hashemi et al. [9] designed an 8.5 cm long single DOF absorber that was able to reduce the tremor at the shoulder and elbow joint from 3° to 0.5° and from 4° to 2.2° in the time domain, respectively. The absorber was said to be tuned, but the natural frequency of the absorber was 2.775 Hz which is different from the system at 2.24 Hz. Rahnavard et al. [10] excited the two DOF hand model using a random input of two different types instead of the sinusoidal excitation. A single DOF tuned absorber was used to reduce the resting tremor with a 20 cm long beam. The absorber was very effective and reduces 60% and 39% of flexion motion due to the first excitation type and 33% and 50% due to the second type in time domain at the shoulder and elbow joints, respectively. In the current work, 9 cm and 7.5 cm long single DOF absorbers are tuned to the first and the second natural frequencies, respectively. The performance of each absorber is studied alone when attached to the forearm and combined to form the “dual DVA” which is tuned at both frequencies. The “dual DVA” is a very effective absorber which can produce a pseudo-steady tremor at hand joints. The absorbers are designed by considering their safety factor against failure by fatigue and yielding. The effect of the damping coefficient of beam absorbers is studied in terms of performance of the absorber in reducing the involuntary tremor and its effect on the safety factors which can reflect its expected period of operation. The absorbers are well designed and prepared for experimental testing on a real human hand.

## 2. System Design

### 2.1. Hand Model

**Figure 1:**
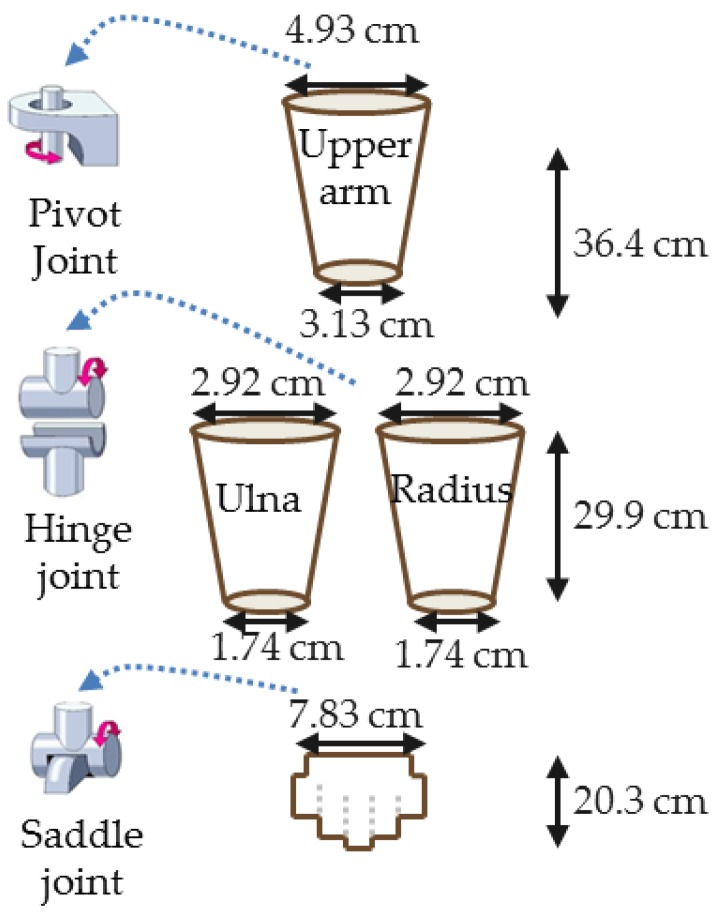
Modeled geometry of the upper arm, forearm and the palm and their corresponding modeled joints.

The human hand is modeled on the horizontal plane as three DOF rigid segments to describe the biodynamic behavior of the upper arm, forearm and the palm. The dimensions of the modeled hand segments and the used one DOF frictionless joints are shown in Figure 1. The upper arm is pinned to the fixed trunk by shoulder joint, the upper arm is hinged to the ulna and radius of the forearm by shoulder joint and the wrist joint connecting the forearm to the palm is modeled as a saddle joint. The right hand parameters are based on the experimentally determined values of length, mass, density and position of centroid demonstrated by Drillis et al. [13]. The mass of the upper arm, forearm and the palm are 2.070 kg, 1.160 kg and 0.540 kg [13], respectively. These values with the designed geometry are used to calculate their mass moments of inertia:

(1) $$I_{1}=0.0228\;kgm^{2}/rd,\;I_{2}=0.0082\;kgm^{2}/rd\;and\;I_{4}=0.0012\;kgm^{2}/rd$$

**Figure 2:**
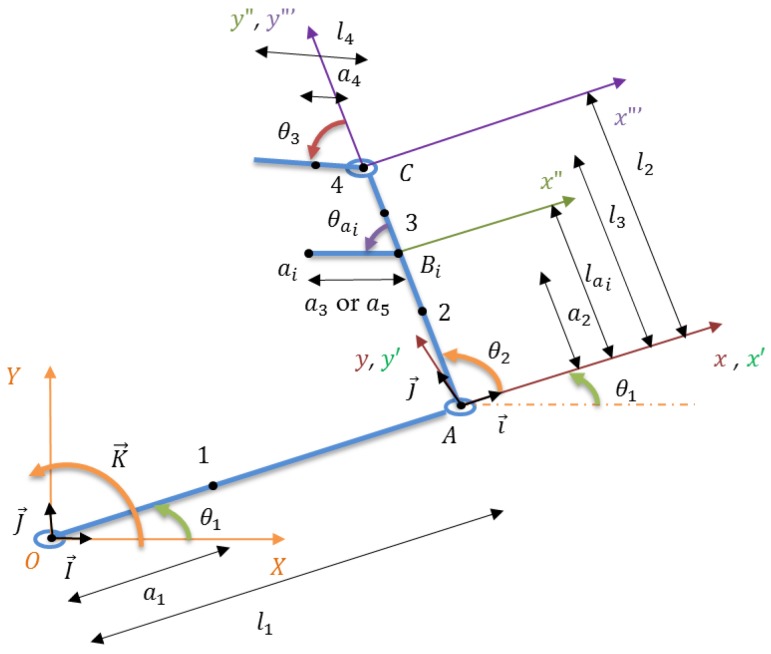
Dynamic modeling of the human hand.

Human hand is modeled to describe the flexion motion at shoulder, elbow and wrist joint. The mass is concentrated at the centroid of each segment. The kinematics and dynamics are determined using the effective hand modeled linkages with the absorber at the forearm (Figure 2).

**Figure 3:**
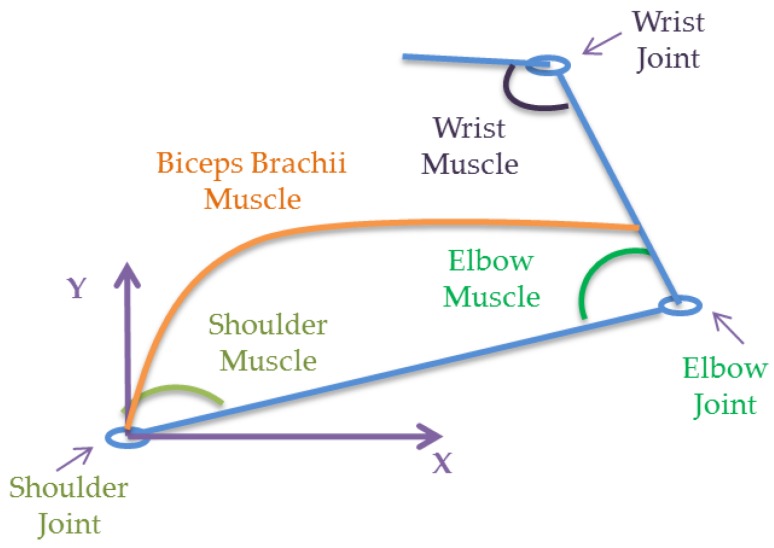
Representation of the hand at musculoskeletal level.

Four muscles are modeled as spring-damper systems in order to produce movement and are assumed to be regulated independently. The single joints shoulder, elbow and wrist joint muscles and the double joint biceps brachii muscle which pairs at the shoulder and elbow are shown in Figure 3. Their stiffness and damping coefficients are assumed to be proportional by a constant as shown in Table 1.

### 2.2. Equations of Motion

Motion will start at an instance of stability when $\theta_{1}=0{}^{\circ},\;\theta_{2}=90{}^{\circ}\;\text{and}\;\theta_{a}=0{}^{\circ}$ with zero angular velocities. This assumption has no effect on the steady state response; it affects only the homogenous response which will disappear after 10 s (determined numerically using the fourth order Runge–Kutta iterative method). At this instance, the kinematics are determined using Coriolis theorem for the five DOF system (using dual DVA) described by five frames rotating with respect to the global coordinate system:

(2) $${\vec{v}}_{1}={\vec{v}}_{O}+{\vec{\Omega }}_{xyz/XYZ}\times {\vec{r}}_{1/O}=a_{1}{\dot{\theta }}_{1}\vec{j}$$

(3) $${\vec{v}}_{A}={\vec{v}}_{O}+{\vec{\Omega }}_{xyz/XYZ}\times {\vec{r}}_{A/O}=l_{1}{\dot{\theta }}_{1}\vec{j}$$

(4) $${\vec{v}}_{2}={\vec{v}}_{A}+{\left({\vec{v}}_{2/A}\right)}_{xyz}+{\vec{\Omega }}_{xyz/XYZ}\times {\vec{r}}_{2/A}=-a_{2}\left({\dot{\theta }}_{1}+{\dot{\theta }}_{2}\right)\vec{i\;}+l_{1}{\dot{\theta }}_{1}\vec{j}$$

(5) $${\vec{v}}_{3}={\vec{v}}_{A}+{\left({\vec{v}}_{3/A}\right)}_{xyz}+{\vec{\Omega }}_{xyz/XYZ}\times {\vec{r}}_{3/A}=-l_{3}\left({\dot{\theta }}_{1}+{\dot{\theta }}_{2}\right)\vec{i\;}+l_{1}{\dot{\theta }}_{1}\vec{j}$$

(6) $${\vec{v}}_{C}={\vec{v}}_{A}+{\left({\vec{v}}_{C/A}\right)}_{xyz}+{\vec{\Omega }}_{xyz/XYZ}\times {\vec{r}}_{C/A}=-l_{2}\left({\dot{\theta }}_{1}+{\dot{\theta }}_{2}\right)\vec{i\;}+l_{1}{\dot{\theta }}_{1}\vec{j}$$

(7) $${\vec{v}}_{4}={\vec{v}}_{C}+{\left({\vec{v}}_{4/C}\right)}_{\overset{´}{x}\overset{´}{y}\overset{´}{z}}+{\vec{\Omega }}_{\overset{´}{x}\overset{´}{y}\overset{´}{z}/XYZ}\times {\vec{r}}_{4/C}=-\left[\left(a_{4}+l_{2}\right)\left({\dot{\theta }}_{1}+{\dot{\theta }}_{2}\right)+a_{4}{\dot{\theta }}_{4}\right]\vec{i\;}+l_{1}{\dot{\theta }}_{1}\vec{j}$$

(8) $${\vec{v}}_{a_{1}}={\vec{v}}_{B_{1}}+{\left({\vec{v}}_{a_{1}/B_{1}}\right)}_{\overset{´}{x}\overset{´}{y}\overset{´}{z}}+{\vec{\Omega }}_{\overset{´}{x}\overset{´}{y}\overset{´}{z}/XYZ}\times {\vec{r}}_{a_{1}/B_{1}}=-\left[\left(l_{a_{1}}+a_{3}\right)\left({\dot{\theta }}_{1}+{\dot{\theta }}_{2}\right)+a_{3}{\dot{\theta }}_{a_{1}}\right]\vec{i\;}+l_{1}{\dot{\theta }}_{1}\vec{j}$$

(9) $${\vec{v}}_{a_{2}}={\vec{v}}_{B_{2}}+{\left({\vec{v}}_{a_{2}/B_{2}}\right)}_{\overset{´}{x}\overset{´}{y}\overset{´}{z}}+{\vec{\Omega }}_{\overset{´}{x}\overset{´}{y}\overset{´}{z}/XYZ}\times {\vec{r}}_{a_{2}/B_{2}}=-\left[\left(l_{a_{2}}+a_{5}\right)\left({\dot{\theta }}_{1}+{\dot{\theta }}_{2}\right)+a_{5}{\dot{\theta }}_{a_{2}}\right]\vec{i\;}+l_{1}{\dot{\theta }}_{1}\vec{j}$$

Equations of motion for the non-Lagrangian system can be derived. The Rayleigh dissipation function is induced within the generalized moments of Lagrange equation to form the equations of motion for the dissipative systems:

(10) $$\frac{d}{dt}\left(\frac{\partial T}{\partial {\dot{q}}_{i}}-\frac{\partial U}{\partial {\dot{q}}_{i}}\right)-\frac{\partial T}{\partial q_{i}}+\frac{\partial U}{\partial q_{i}}=F_{i}+F_{c_{i}},\;i=\left\{1,2,3,a_{1},a_{2}\right\}$$

(11) $$F_{c_{i}}=-\frac{\partial R}{\partial {\dot{q}}_{i}},\;with\;R_{i}=\frac{1}{2}c{\dot{q}}_{i}^{2}$$

- Kinetic Energy
  (12) $$T=\left[\frac{1}{2}I_{1}{\dot{\theta }}_{1}^{2}+\frac{1}{2}m_{1}v_{1}^{2}\right]+\left[\frac{1}{2}I_{2}{\left({\dot{\theta }}_{1}+{\dot{\theta }}_{2}\right)}^{2}+\frac{1}{2}m_{2}v_{2}^{2}\right]+\frac{1}{2}m_{3}v_{3}^{2}+\left[\frac{1}{2}I_{4}{\dot{\theta }}_{4}^{2}+\frac{1}{2}m_{4}v_{4}^{2}\right]+\frac{1}{2}m_{a_{1}}v_{a_{1}}^{2}+\frac{1}{2}m_{a_{2}}v_{a_{2}}^{2}$$
- Potential Energy
  (13) $$U=\frac{1}{2}k_{1}\theta_{1}^{2}+\frac{1}{2}k_{2}\theta_{2}^{2}+\frac{1}{2}k_{3}{\left(\theta_{1}+\theta_{2}\right)}^{2}+\frac{1}{2}k_{4}\theta_{3}^{2}+\frac{1}{2}k_{a_{1}}\theta_{a_{1}}^{2}+\frac{1}{2}k_{a_{2}}\theta_{a_{2}}^{2}$$
- Rayleigh dissipation function
  (14) $$R=\frac{1}{2}c_{1}{\dot{\theta }}_{1}^{2}+\frac{1}{2}c_{2}{\dot{\theta }}_{2}^{2}+\frac{1}{2}c_{3}{\left({\dot{\theta }}_{1}+{\dot{\theta }}_{2}\right)}^{2}+\frac{1}{2}c_{4}{\dot{\theta }}_{3}^{2}+\frac{1}{2}c_{a_{1}}{\dot{\theta }}_{a_{1}}^{2}+\frac{1}{2}c_{a_{2}}{\dot{\theta }}_{a_{2}}^{2}$$

Then, the generalized equation of motion has the form:

(15) $$\left[M\right]\left\{\ddot{\theta }\right\}+\left[C\right]\left\{\dot{\theta }\right\}+\left[K\right]\left\{\theta \right\}=\left\{f\right\}$$

where,

(16) $$\left[M\right]=\left[\begin{array}{ccccc} M_{11} & M_{12} & M_{13} & M_{14} & M_{15}\\ M_{21} & M_{22} & M_{23} & M_{24} & M_{25}\\ M_{31} & M_{32} & M_{33} & M_{34} & M_{35}\\ M_{41} & M_{42} & M_{43} & M_{44} & M_{45}\\ M_{51} & M_{52} & M_{53} & M_{54} & M_{55} \end{array}\right]\;M_{11}=\left(I_{1}+m_{1}a_{1}^{2}\right)+\left(I_{2}+m_{2}a_{2}^{2}\right)+m_{2}l_{1}^{2}+m_{3}\left(l_{1}^{2}+l_{3}^{2}\right)+m_{4}\left(l_{1}^{2}+l_{2}^{2}+a_{4}^{2}+2l_{2}a_{4}\right)\;+m_{a_{1}}\left(l_{1}^{2}+l_{a_{1}}^{2}+a_{3}^{2}+2l_{a_{1}}a_{3}\right)+m_{a_{2}}\left(l_{1}^{2}+l_{a_{2}}^{2}+a_{5}^{2}+2l_{a_{2}}a_{5}\right)\;M_{12}=\left(I_{2}+m_{2}a_{2}^{2}\right)+m_{3}l_{3}^{2}+m_{4}\left(l_{2}^{2}+a_{4}^{2}+2l_{2}a_{4}\right)+m_{a_{1}}\left(l_{a_{1}}^{2}+a_{3}^{2}+2l_{a_{1}}a_{3}\right)+m_{a_{2}}\left(l_{a_{2}}^{2}+a_{5}^{2}+2l_{a_{2}}a_{5}\right)\;M_{13}=m_{4}\left(a_{4}^{2}+l_{2}a_{4}\right),\;M_{14}=m_{a_{1}}\left(a_{3}^{2}+l_{a_{1}}a_{3}\right),\;M_{15}=m_{a_{2}}\left(a_{5}^{2}+l_{a_{2}}a_{5}\right)\;M_{21}=M_{12},\;M_{22}=M_{12},\;M_{23}=M_{13},\;M_{24}=M_{14},\;M_{25}=M_{15}\;M_{31}=M_{13},\;M_{32}=M_{23},\;M_{33}=I_{4}+m_{4}a_{4}^{2},\;M_{34}=0,\;M_{35}=0\;M_{41}=M_{14},\;M_{42}=M_{24},\;M_{43}=M_{34},\;M_{44}=m_{a_{1}}a_{3}^{2},\;M_{45}=0\;M_{51}=M_{15},\;M_{52}=M_{25},\;M_{53}=M_{35},\;M_{54}=M_{45},\;M_{55}=m_{a_{2}}a_{5}^{2}$$

(17) $$\left[K\right]=\left[\begin{array}{ccccc} k_{1}+k_{3} & k_{3} & 0 & 0 & 0\\ k_{3} & k_{2}+k_{3} & 0 & 0 & 0\\ 0 & 0 & k_{4} & 0 & 0\\ 0 & 0 & 0 & k_{a_{1}} & 0\\ 0 & 0 & 0 & 0 & k_{a_{2}} \end{array}\right]$$

(18) $$\left[C\right]=\left[\begin{array}{ccccc} c_{1}+c_{3} & c_{3} & 0 & 0 & 0\\ c_{3} & c_{2}+c_{3} & 0 & 0 & 0\\ 0 & 0 & c_{4} & 0 & 0\\ 0 & 0 & 0 & c_{a_{1}} & 0\\ 0 & 0 & 0 & 0 & c_{a_{2}} \end{array}\right]$$

(19) $$f={\left\{\begin{array}{ccccc} f_{1} & f_{2} & 0 & 0 & 0 \end{array}\right\}}^{T}$$

Input moments to the model are considered as muscular activity due to shoulder and elbow muscles’ activation to see their effect on the motion transmitted to the palm. The same equations are obtained for the primary system and systems controlled by single absorbers, but with some simplifications.

The natural frequencies of the primary system are obtained to be in range of pathological tremor using the system’s characteristic equation:

(20) $$\omega_{n_{1}}=3.564\;Hz,\;\omega_{n_{2}}=5.296\;Hz\;and\;\omega_{n_{3}}=12.496\;Hz$$

### 2.3. Absorber Design

**Figure 4:**
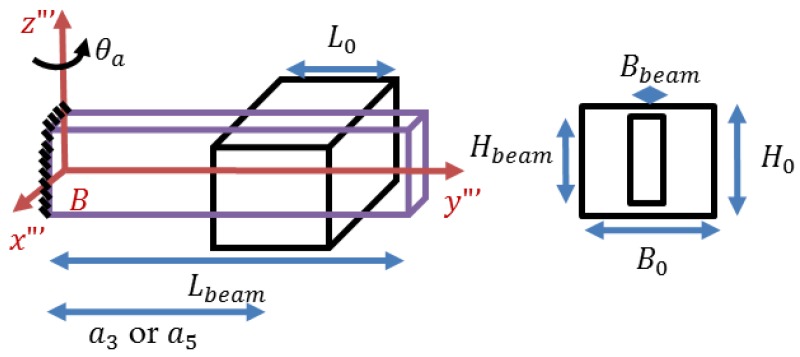
Designed dynamic vibration absorber.

The absorber is modeled as a stainless steel alloy cantilevered beam with a copper mass attached along its length as in Figure 4. Two absorbers are designed to be attached on the forearm.

The dimensions of “absorber 1” and “absorber 2” systems are shown in Table 2. The effect of using each absorber alone is compared to the response of the hand when combining both absorbers together which are attached at the same position on the forearm to form the ”dual DVA”. The absorbers are considered with an additional 52 g, respresenting the mass of the controller device.

The natural frequency of the absorber’s system is approximated using Dunkerley’s formulation which gives a lower band approximation [14]:

(21) $$\frac{1}{\omega_{a}^{2}}≃\frac{1}{\omega_{beam}^{2}}+\frac{1}{\omega_{m_{0}}^{2}}$$

where,

(22) $$\omega_{beam}=3.5160\sqrt{\frac{E_{beam}I_{beam}}{m_{beam}L_{beam}^{3}}}$$

(23) $$\omega_{m_{0}}=\sqrt{\frac{6E_{beam}I_{beam}}{m_{0}a_{i}^{2}\left(3L_{beam}-a_{i}\right)}},\;i=\left\{3,5\right\}$$

(24) $$E_{beam}=189.6\;\text{GP},\;\rho_{beam}=7800\;kg/m^{3}\;and\;\rho_{m_{0}}=8900\;kg/m^{3}$$

Hence,

(25) $$a_{3}=7.76\;cm\;and\;a_{5}=5.31\;cm\;$$

## 3. Results and Discussions

### 3.1. Complex Transfer Function

The behavior at the proximal joints of the dynamically coupled modeled system can be determined by solving the system’s equation of motion. The response is determined using the complex transfer function of the system:

(26) $$H\left(\omega \right)={\left\{\left(-\omega^{2}\left[M\right]+\left[K\right]\right)+j\omega \left[C\right]\right\}}^{-1}$$

Each input moment due to shoulder and elbow muscles’ activation is assumed to have the same magnitude. Both inputs are assumed to behave as sinusoidal functions to reflect the rhythmic motion of hand tremor driven at resonance. Muscles can operate in a range of driving frequencies, but only the critical frequencies in the range of resting tremor will be considered:

(27) $$f_{k}=F_{k_{1}}\cos\left(\omega_{1}t\right)+F_{k_{2}}\cos\left(\omega_{2}t\right),\;k=\left\{1,2\right\}\;F_{k_{1}}=F_{k_{2}}=1N.m\;and\;\omega_{m}=\omega_{n_{m}},\;m=\left\{1,2\right\}$$

The response depends on the Reacceptance function of the form:

(28) $$\alpha_{k}=\left\{\begin{array}{c} \frac{A_{1_{ik}}+jB_{1_{ik}}}{A_{2}+jB_{2}}\\ \vdots \\ \frac{A_{1_{nk}}+jB_{1_{Nk}}}{A_{2}+jB_{2}} \end{array}\right\},\;i=\left\{1,\ldots ,5\right\}\;and\;n=\left\{1,\ldots ,5\right\}$$

The response is the sum of responses due to each excitation moment. Then, the response in the frequency domain can be determined to analyze the behavior of the system over a range of driving frequencies.

(29) $$\Theta =\overset{2}{\underset{k=1}{\sum }}\overset{2}{\underset{m=1}{\sum }}\alpha_{k}F_{k_{m}}$$

The time domain response is also examined, in order to analyze its behavior at the specified excitation frequencies.

(30) $$\theta_{ik}=\left|\Theta_{ik}\right|e^{j\left(\omega_{m}t-\varphi \right)},\;\varphi =\tan^{-1}\left(\frac{B_{1_{ik}}}{A_{1_{ik}}}\right)$$

The response serves in designing the unknown absorber’s parameters. Each absorber is tuned from the root of the real part in the numerator of the chosen response, and then the stiffness of the absorber can be determined. The damping coefficient of each absorber is assumed to be proportional to its stiffness coefficient by a constant.

Although there is no damper added to the absorber, very little damping will be provided from the beam’s material. However, the damping coefficient must be increased to maintain low amplitudes of oscillation in the absorber in order to avoid impact with the forearm. So, the beam material can be coated by damping material to increase its value to:

(31) $$c_{a_{1}}=0.0002k_{a_{1}}\;and\;c_{a_{2}}=0.0002k_{a_{2}}$$

“Absorber 1” is designed to be tuned at the wrist joint’s response $\Theta_{31}$ with the first resonance frequency $\omega_{n_{1}}$, due to shoulder muscle activation $F_{1_{1}}$.

(32) $$A_{1_{31}}=-\omega^{6}\left(M_{21}M_{32}M_{44}-M_{22}M_{32}M_{44}\right)+\omega^{4}\left(-M_{32}M_{44}k_{2}+M_{21}M_{32}k_{a}-M_{22}M_{32}k_{a}-M_{32}c_{2}c_{a}\right)+\omega^{2}\left(M_{32}k_{2}k_{a}\right)$$

“Absorber 2” is designed to be tuned at the wrist joint’s response $\Theta_{32}$ with the second resonance frequency $\omega_{n_{2}}$, due to elbow muscle activation $F_{2_{2}}$.

(33) $$A_{1_{32}}=\omega^{6}\left(M_{11}M_{32}M_{44}-M_{12}M_{32}M_{44}\right)-\omega^{4}\left(M_{32}M_{44}k_{1}+M_{11}M_{32}k_{a}-M_{12}M_{32}k_{a}+M_{32}c_{1}c_{a}\right)+\omega^{2}\left(M_{32}k_{1}k_{a}\right)$$

Solving (32) and (33), then all absorbers’ parameters are determined (Table 3) to satisfy the tuning conditions of “absorber 1” and “absorber 2”, respectively:

(34) $$A_{1_{31}}=0\;and\;\omega_{a_{1}}=\omega_{n_{1}}$$

(35) $$A_{1_{32}}=0\;and\;\omega_{a_{2}}=\omega_{n_{2}}$$

**Figure 5:**
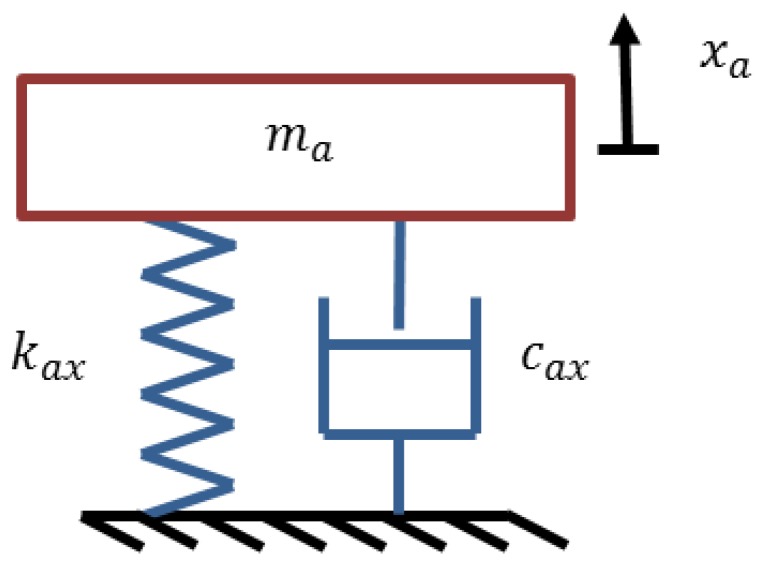
Equivalent linear model of the pendulum absorber.

Each modeled pendulum absorber can have its equivalent conventional mass-linear spring and damper system as shown in Figure 5.

These pendulum absorbers can be also attached to the forearm in the form of their equivalent models with their corresponding parameters as shown in Table 4.

(36) $$x_{a_{1}}≃a_{3}\theta_{a_{1}}\;and\;x_{a_{2}}≃a_{5}\theta_{a_{2}}$$

(37) $$k_{a_{1x}}≃\frac{k_{a_{1}}}{a_{3}^{2}}\;and\;k_{a_{2x}}≃\frac{k_{a_{2}}}{a_{5}^{2}}$$

Steel beams have a very low damping ratio provided from its material [7]. The damping ratio (ζ) of a steel bar structure varies between 0.001 and 0.002 without adding an external damper. The mixed damping ratio of “absorber 1” (*i* = 1) and “absorber 2” (*i* = 2) can be calculated according to the following equation:

(38) $$\zeta_{a_{i}}=\frac{c_{a_{xi}}}{2M_{a_{i}}\omega_{n_{i}}},\;i=\left\{1,\;2\right\}$$

Substitute the calculated absorber’s parameters into (38) to obtain the mixed damping ratio of “absorber 1” and “absorber 2”, respectively, as 0.0015 and 0.0020. These damping ratios are within their accepted range. $\omega_{n_{i}}$ is the natural frequency of the primary system. 

### 3.2. Numerical Simulations

Results are obtained using the MATLAB program in order to show the behavior of the system in the time and frequency domains. Graphs will show the behavior of the system due to shoulder and elbow muscle activation. The palm is the main organ of the hand which is responsible for human grasping and non-grasping activities. So, the absorbers are tuned at the wrist joint’s response to have high suppression at the palm. The time and frequency domain responses at the hand joints are shown for the uncontrolled system and compared to that of the system controlled by different passive absorbers. The performance of “absorber 1” and “absorber 2”, each tuned to different driving frequencies, will be compared to that of the “dual DVA” which is the combination of both absorbers under the same controller device. The performance in the time domain will be analyzed in terms of the absorber’s capability in reducing tremor amplitude, using:

(39) $$\%\;Reduction=\frac{\Theta_{uncontrolled}-\Theta_{controlled}}{\Theta_{uncontrolled}}\times 100$$

#### 3.2.1. Behavior in Frequency Domain

**Figure 6:**
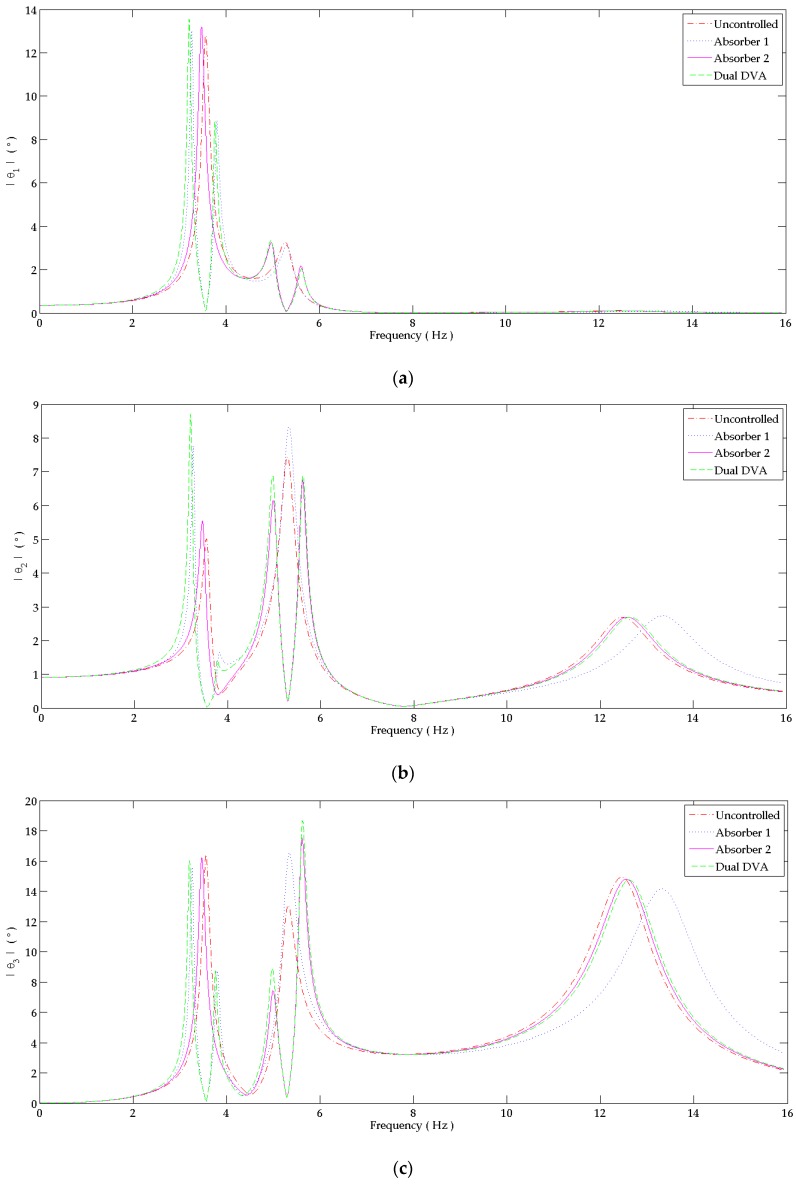
Response in the frequency domain at: (**a**) Shoulder joint; (**b**) Elbow joint; (**c**) Wrist joint. The behavior at each joint is studied when attaching to the forearm: “absorber 1” alone, “absorber 2” alone and the ”dual DVA” tuned at wrist joint’s responses. It is compared to the response of the uncontrolled system.

In Figure 6a–c, the frequency domain response of the shoulder, elbow and wrist joints are shown. Tuning the absorbers causes a high reduction in tremor amplitude at the tuned natural frequency of the primary system. The controlled system’s natural frequency is shifted to the right and the left of this tuning point decreasing its amplitude neighborhood zero. The damped tremor’s amplitude at the tuning point depends on the values of the absorber’s damping coefficient. The involuntary flexion motion at the proximal joint’s response of Figure 6 shows the behavior of the different tuned absorbers. The tuning condition of “absorber 1” of (34) is well shown at the primary system’s fundamental frequency only, while “absorber 2” shows tuning point at the second natural frequency only according to (35). The “dual DVA” is tuned to the first two resonance frequencies due to (34) and (35). Its behavior at these frequencies is well represented at the primary system’s first two peaks of all hand joints where very low amplitudes are shown. So, the combination of “absorber 1” and “absorber 2” results in the improvement of the absorber’s behavior for a system tuned at more than one resonance frequency.

The shifted natural frequencies around the tuning frequencies of “absorber 1”, “absorber 2” and the “dual DVA” can be recognized when comparing them with the uncontrolled systems’ natural frequencies (20). The natural frequencies are:

- For the four DOF system with “absorber 1”:
  (40) $$\omega_{n_{1}}=3.267\;Hz,\;\omega_{n_{2}}=3.800\;Hz,\;\omega_{n_{3}}=5.329\;Hz\;and\;\omega_{n_{4}}=13.366\;Hz\;$$
- For the four DOF system with “absorber 2”:
  (41) $$\omega_{n_{1}}=3.474\;Hz,\;\omega_{n_{2}}=5.001\;Hz,\;\omega_{n_{3}}=5.603\;Hz\;and\;\omega_{n_{4}}=12.596\;Hz$$
- For the five DOF system with the “dual DVA”:
  (42) $$\omega_{n_{1}}=3.211\;Hz,\;\omega_{n_{2}}=3.760\;Hz,\;\omega_{n_{3}}=4.986\;Hz,\;\omega_{n_{4}}=5.614\;Hz\;and\;\omega_{n_{5}}=12.656\;Hz$$

**Figure 7:**
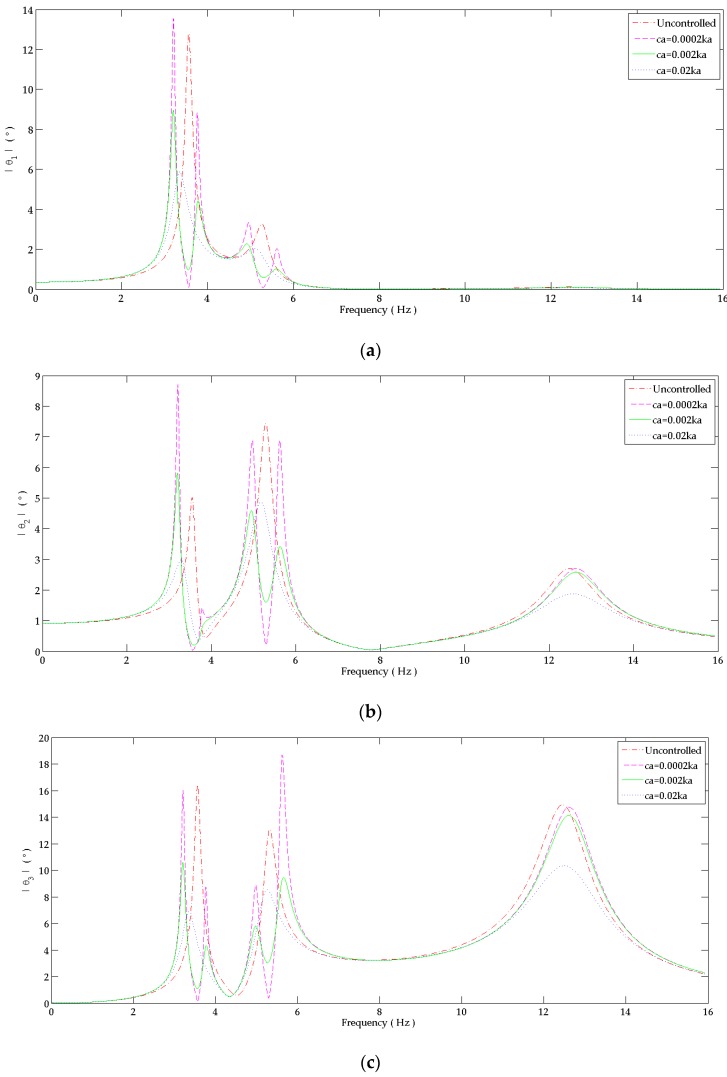
Response in the frequency domain at: (**a**) Shoulder joint; (**b**) Elbow joint; (**c**) Wrist joint. The behavior at each joint is studied when attaching to the forearm due to the ”dual DVA” tuned at wrist joint’s responses. It is compared to the response of the uncontrolled system.

The effect of absorber’s damping coefficient in reducing tremor amplitude is presented in Figure 7 for the hand controlled by the ”dual DVA”. The damping coefficient is an important parameter to be considered. The peaks corresponding to the shifted frequencies around the tuning conditions of (34) and (35) decrease as the damping coefficient increases from 0.0002$k_{a}$ to 0.02$k_{a}$ (Figure 7a–c). When using a high absorber’s damping coefficient of 0.02$k_{a}$, the shifted peaks are highly damped to produce three peaks instead of the five peaks that must be shown at the five resonance frequencies. It avoids the critical vibrations around the tuning frequencies but leaving high amplitudes at these frequencies at the tuning points in comparison to the light damping absorbers. On the other hand, the magnitude of the flexion angle at the three peaks of the hand controlled by the dual absorber with $c_{a}$ = 0.02$k_{a}$ is less than the primary system’s corresponding peaks. In order to have an absorber with a high damping coefficient, the surface of the absorber’s beam surface can be coated by a highly damped material.

#### 3.2.2. Behavior in Time Domain

**Figure 8:**
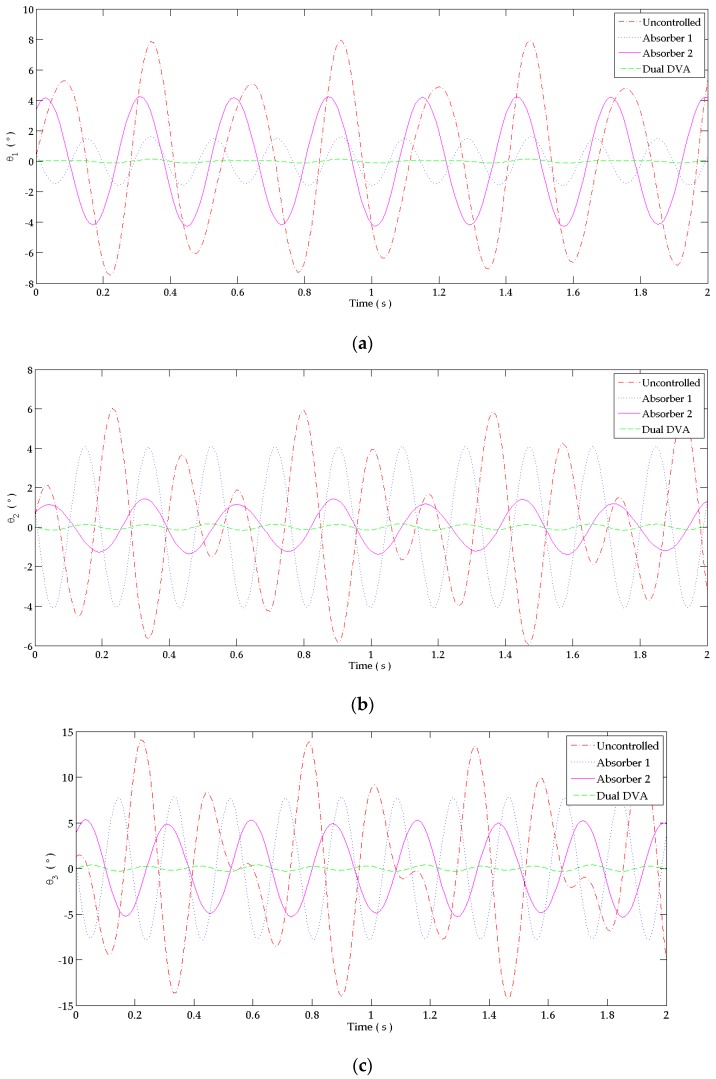
Response in the time domain at: (**a**) Shoulder joint; (**b**) Elbow joint; (**c**) Wrist joint. The behavior at each joint is studied when attaching to the forearm: “absorber 1” alone, “absorber 2” alone and the “dual DVA” tuned at wrist joint’s responses. It is compared to the response of the uncontrolled system.

The human hand system is excited at the first and second resonance frequencies of the primary system reflecting the critical operating frequencies due to shoulder and elbow muscle activation. “Dual DVA” is used to satisfy the tuning conditions at both frequencies. The response of the uncontrolled and controlled systems is shown in Figure 8a–c. It is shown that all designed absorbers reduced the flexion motion at the joints. The ”dual DVA” was more efficient than “absorber 1” and “absorber 2” in suppressing involuntary tremor at the shoulder, elbow and wrist joints in the time domain.

The percentage of reduction of the response in the time domain due to attaching each absorber is shown in Table 5 when using an absorber with a 0.0002$k_{a}$ damping coefficient of a steel bar having a damping ratio between 0.001 to 0.002 [15]. The percentage of reduction is calculated using (39). The “dual DVA” leads to the best reduction since its damping coefficient approaches zero to reflect the behavior of the thin steel material having negligible damping. “Absorber 1” and “absorber 2” alone can cause a considerable reduction in tremor amplitude at the three joints even though they are tuned at a single frequency. Tuning both absorbers together for the “Dual DVA” causes a pseudo-steady tremor at hand joints. The “dual DVA” absorber reduces about 98.3%–99.5%, 97.0%–97.3% and 97.4%–97.5% of the shoulder, elbow and wrist joint’s responses, respectively for $k_{a}$ = 0.0002$k_{a}$.

**Figure 9:**
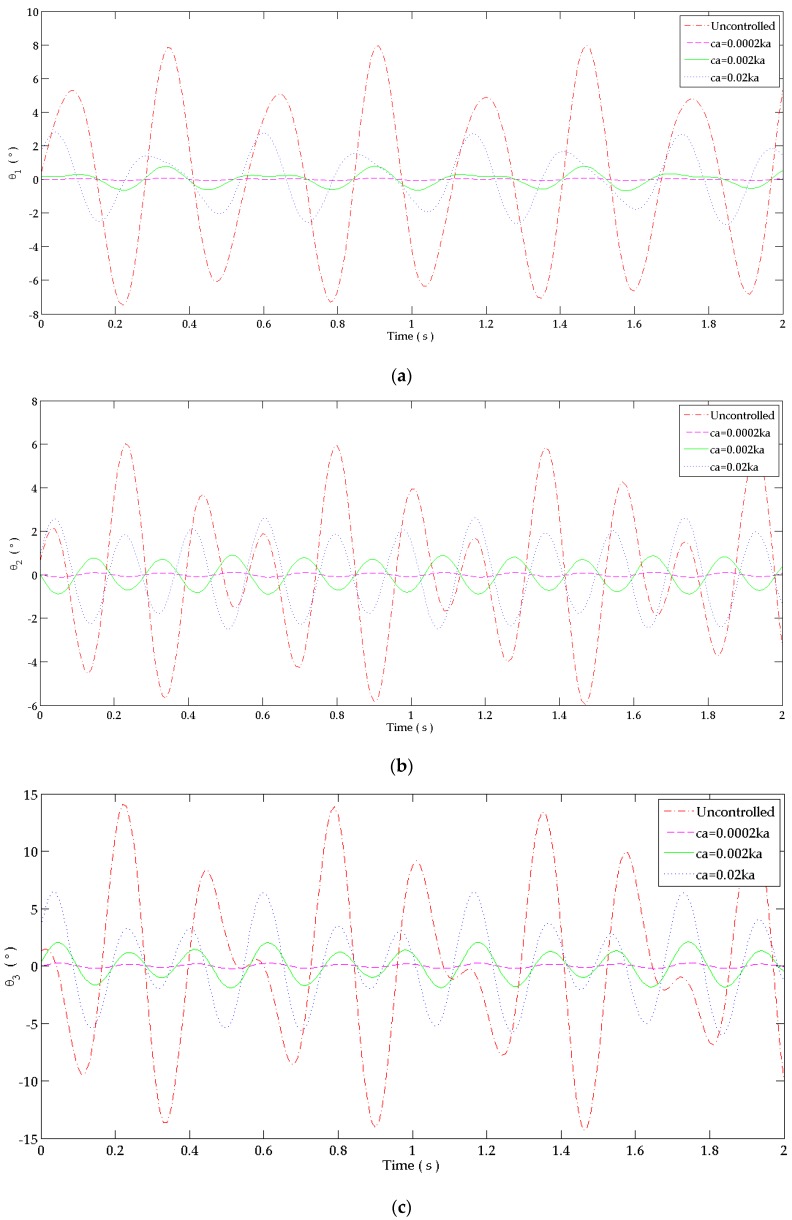
Response in the frequency domain at: (**a**) Shoulder joint; (**b**) Elbow joint; (**c**) Wrist joint. The behavior at each joint is studied when attaching to the forearm due to the ”dual DVA” tuned at wrist joint’s responses. It is compared to the response of the uncontrolled system.

The effect of the damping coefficient $\left(c_{a}=\left\{0.0002k_{a},0.002k_{a},0.02k_{a}\right\}\right)$ of both steel beams absorbers is shown in Figure 9a–c for the ”dual DVA” in the time domain. It is shown that the maximum amplitude of the flexion angle at shoulder, elbow and wrist joints decreases as the damping coefficient value increases.

The percentage of reduction which results due to each tested damping coefficient in the time domain of Figure 9 is calculated using (39) and is shown in Table 6. As the damping coefficients of the absorber increases, the percentage of reduction of the tremor’s amplitude decreases. For a high damping coefficient, the “dual DVA” tuned at the first two natural frequencies (24) and (35) can also cause a good reduction of the primary system’s amplitude when coating the beam’s surface by a damping material to increase its damping coefficient without adding a damper. The 0.02$k_{a}$ steel bar can reduce 65.24%–68.2%, 49.7%–57.1% and 54.1%–60.52% of the tremor’s amplitude at shoulder, elbow and wrist joints, respectively.

### 3.3. Absorber’s Lifetime

**Figure 10:**
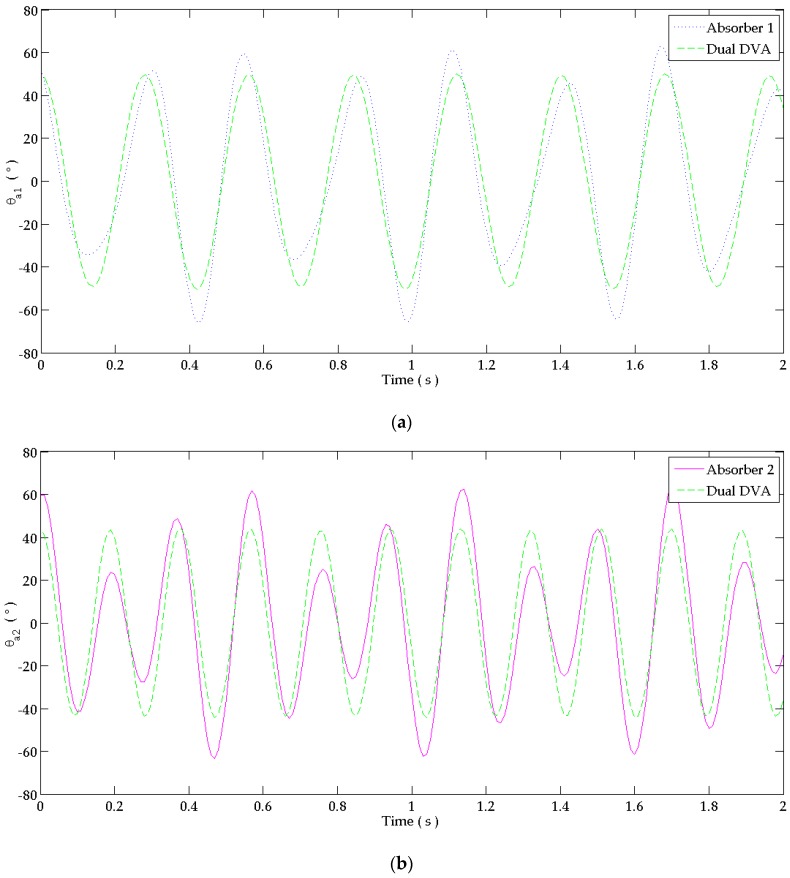
Response in time domain at: (**a**) “absorber 1”; (**b**) “absorber 2” joints. The fluctuations at the joints of the absorbers when attached alone and together as a ”dual DVA” for $c_{a}$ = 0.0002$k_{a}$.

The period of operation of the designed absorber can be theoretically calculated to know the estimated number of cycles before its failure by fatigue. This calculation gives information about the designed dimensions and chosen materials of the absorber if they are appropriate to bear these fluctuations. Figure 10a and b shows the behavior of “absorber 1” and “absorber 2” when attached separately to the primary system and their behavior when attached together as a “dual DVA” in the time domain for a 0.0002$k_{a}$ damping coefficient each.

The absolute maximum and minimum angular displacement for each absorber of Figure 10 are summarized in Table 7 for a damping coefficient of 0.0002$k_{a}$. The fluctuation angle of the cantilevered beam absorbers is an important parameter needed to calculate the acting bending stress which will identify how critical its vibration is. For the system excited at the first two natural frequencies, the absorbers oscillate between 43° and 61.8° approximately.

The cold rolled stainless steel alloy (type 301) is selected as beams material of both absorbers $\left(S_{ut}=1379\;MPa\;and\;S_{y}=1138\;MPa\right)$ and assumed to have 90% reliability and tested at room temperature. The endurance limit of the selected material can be calculated after multiplying it by correction factors to reflect the endurance limit of the specimen used in a rotating beam experiment test. Then, the safety factors against failure by fatigue based on the Modified Goodman diagram and yielding of the beam can be calculated according to the following equations:

(43) $$\sigma =\frac{{ℳ}_{a}\frac{B_{0}}{2}}{I_{beam}},\;Where\;{ℳ}_{a}=k_{a}\Theta_{a}$$

(44) $$S_{e}^{\prime }=0.5S_{ut}\;\left(S_{ut}>1400\;MPa\right)$$

(45) $$S_{e}=C_{load}C_{size}C_{surf}C_{temp}C_{reliab}S_{e}^{\prime }$$

(46) $$N_{f}=\frac{S_{e}S_{ut}}{\sigma_{alt.}^{\prime }S_{ut}+\sigma_{mean}^{\prime }S_{e}}$$

(47) $$N_{y}=\frac{S_{y}}{\sigma_{max}}$$

The corrected endurance limit of the absorbers’ material is calculated to be $S_{e}=410.67\;MPa$. Then, the obtained safety factors for each of “absorber 1”, “absorber 2” and their combination in the “dual DVA” are shown in Table 8. “Absorber 1” is safe against failure but its safety factor is approaching 1. Critical oscillations are transmitted to “absorber 2” and it might not operate for a longer period. The combination of both absorbers is safer than using each absorber alone $\left(N_{f}>1\;and\;N_{y}>1\right)$ due to the lower fluctuating angular displacements resulting on this “dual DVA” absorber as shown in Table 7.

**Figure 11:**
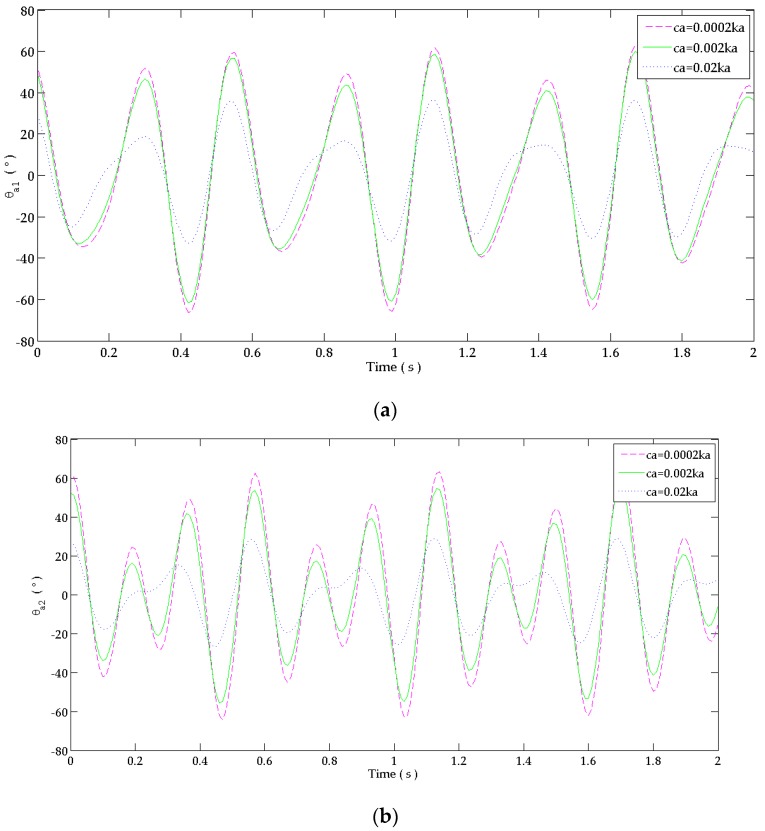
Response in time domain at: (**a**) “absorber 1”; (**b**) “absorber 2” joints. The fluctuation of each absorber when it is attached alone to the forearm for different beams’ damping coefficients.

To obtain a safer design, another stainless steel alloy beam’s material (to maintain the same design) of a higher endurance limit can be selected. So, it will tolerate high bending stresses without changing the performance of the absorber (%reduction). In addition, the beam’s damping coefficient may be increased by coating absorbers surface by a damping material to increase the absorber’s safety. Figure 11 shows the behavior of “absorber 1” and “absorber 2” when attached alone for different beam’s damping coefficients since critical behavior was shown at these absorbers. It is shown that the maximum amplitude of each absorber decreases as the damping coefficient increases.

The maximum and minimum flexion angle of “absorber 1” and “absorber 2” of Figure 11 are summarized in Table 9. It shows that the maximum and minimum angular displacements are decreasing as the damping coefficient is increasing. These values are used to calculate the safety factor of these absorbers.

The safety factors of “absorber 1” and “absorber 2” are shown in Table 10 for different damping coefficients of the beam. As the damping coefficient increases, the absorbers are safer against fatigue and yielding. “Absorber 1” and “absorber 2” of the ”dual DVA” with $c_{a}$ = 0.0002$k_{a}$ can operate for a longer period without fatigue (Table 8) than “absorber 1” and “absorber 2” when each is attached alone with a high damping coefficient of $c_{a}$ = 0.02$k_{a}$, i.e., the ”dual DVA” is safer than a single absorber can operate for a longer time with a high performance. When manufacturing these single absorbers, the damping coefficient of their beams must be increased to ensure that it may operate without failure.

## 4. Conclusions

The human hand was modeled dynamically as a three DOF system to describe the flexion angular motion at the proximal joints in the horizontal plane. The modeled system was able to reflect the biodynamic response of patients suffering from pathological tremor. The system was excited at resonance using two harmonic functions due to each of the shoulder and elbow muscles’ activation, since muscles do not operate at a single and constant frequency. Mechanical treatment using tuned vibration absorbers was suggested to help such neurologically disordered people.

The performance of the single and dual DVA were compared in terms of their capability in reducing the amplitude of the involuntary tremor. Two single DVA were used, each tuned to one of the excitation frequencies. Each absorber alone was able to reduce the tremor’s amplitude at the proximal joints and mainly at the wrist joint to suppress tremor transmitted to the palm. Both single DVAs are combined together under one controller device to form the dual DVA tuned at both driving frequencies. The absorbers were designed respecting their geometric limitations and constraints and by considering patients’ comfort in holding this absorber. The absorber’s device was considered to allow its vibration in the flexion direction only. The fatigue and yielding safety factors were considered within the design. Results show that, as the damping coefficient of the absorber increases, a safer design is obtained but with lower performance. The dual DVA was more efficient than both single absorbers in attenuating the tremor. It can operate for a longer period without failure with a higher percentage of reduction in a tremor’s amplitude. It causes 98.3%–99.5%, 97.0%–97.3% and 97.4%–97.5% reduction in the flexion motion of the primary system at shoulder, elbow and wrist joints. When manufacturing this absorber to be tested experimentally on a real hand, the above percentages might have lower values than the theoretical ones.

As future work, an experimental rig will be fabricated to hold the actual hand and measure the flexion angular displacement at the proximal joints. The absorber with its controller device will be manufactured to be tested on a real human hand for patients suffering from Parkinson’s disease. So, an experiment can be conducted on a real case to validate this semi-analytical approach and to probe any potential “real-life” side effects of the system. In addition, the hand’s DOF can be increased to take into account the forearm pronation-supination at the elbow joint and the wrist joint’s radial-ulnar angular motions. Then, an absorber can be designed to give efficient suppression of the involuntary motion in all modeled directions.

## Figures and Tables

**Table 1:** Stiffness and damping coefficients of the muscles.

Muscle	Shoulder	Elbow	Biceps	Wrist
k (Nm/rd)	180	70	40	10
c (Nms/rd)	0.002$k_{1}$	0.002$k_{2}$	0.002$k_{3}$	0.001$k_{4}$

**Table 2:** Dimensions of the designed absorbers systems.

Dimensions		L (cm)	H (cm)	B (cm)
Absorber 1	Beam 1	9	2.4	0.03
	Attached Mass 1	2.24	2.3	2.3
Absorber 2	Beam 2	7.5	1.8	0.03
	Attached Mass 2	2.4	2	2

**Table 3:** Parameters of the designed pendulum absorbers.

Parameters	$m_{a}$ (g)	$k_{a}$ (Nm/rd)	$c_{a}$ (Nms/rd)
**Absorber 1**	105.239	0.3184	0.0002$k_{a}$
**Absorber 2**	85.044	0.2662	0.0002$k_{a}$

**Table 4:** Parameters of the designed conventional linear absorbers.

Parameters	$m_{a}$ (g)	$k_{ax}$ (N/m)	$c_{ax}$ (Ns/m)
Absorber 1	105.239	52.78	0.0002$k_{ax}$
Absorber 2	85.044	94.11	0.0002$k_{ax}$

**Table 5:** Percentage of reduction in flexion motion at hand joints due to attaching “absorber 1”, “absorber 2” and “dual absorber” in the time domain.

% Reduction	Absorber 1	Absorber 2	Dual DVA
Shoulder	69.1%–79.7%	12.6%–47.2%	98.3%–99.5%
Elbow	4.05%–32.12%	72.6%–76.3%	97.0%–97.3%
Wrist	6.4%–44.2%	40.2%–62.9%	97.4%–97.5%

**Table 6:** Percentage of reduction in flexion motion at hand joints due to the “dual absorber” in the time domain.

% Reduction	$c_{a}$ = 0.0002$k_{a}$	$c_{a}$ = 0.002$k_{a}$	$c_{a}$ = 0.02$k_{a}$
Shoulder	98.3%–99.5%	90.9%–93.9%	65.24%–68.2%
Elbow	97.0%–97.3%	80.8%–85.4%	49.7%–57.1%
Wrist	97.4%–97.5%	85.6%–85.9%	54.1%–60.52%

**Table 7:** Minimum and maximum flexion angle of the absorbers in time domain for $c_{a}$ = 0.0002$k_{a}$.

Flexion Angle	Absorber 1	Absorber 2	Dual DVA	
			Absorber 1	Absorber 2
$\Theta_{min}$	42.97°	23.83°	49.43°	43.00°
$\Theta_{max}$	61.45°	61.81°	49.96°	43.53°

**Table 8:** Fatigue and yielding safety factors of the absorber’s beams for $c_{a}$ = 0.0002$k_{a}$.

Safety Factor	Absorber 1	Absorber 2	Dual DVA	
			Absorber 1	Absorber 2
$N_{f}$	1.07	0.69	1.77	1.64
$N_{y}$	1.20	1.07	1.47	1.54

**Table 9:** Minimum and maximum flexion angle of the absorbers in time domain for different beams’ damping coefficients.

Safety Factor		Absorber 1	Absorber 2
$c_{a}$ = 0.0002$k_{a}$	$\Theta_{min}$	42.97°	23.83°
	$\Theta_{max}$	61.45°	61.81°
$c_{a}$ = 0.002$k_{a}$	$\Theta_{min}$	40.81°	18.69°
	$\Theta_{max}$	58.61°	54.60°
$c_{a}$ = 0.02$k_{a}$	$\Theta_{min}$	14.78°	5.64°
	$\Theta_{max}$	36.16°	28.40°

**Table 10:** Fatigue and yielding safety factors of the absorber’s beams for different beams’ damping coefficients.

Safety Factor		Absorber 1	Absorber 2
$c_{a}$ = 0.0002$k_{a}$	$N_{f}$	1.07	0.69
	$N_{y}$	1.20	1.07
$c_{a}$ = 0.002$k_{a}$	$N_{f}$	1.21	0.73
	$N_{y}$	1.25	1.21
$c_{a}$ = 0.02$k_{a}$	$N_{f}$	1.42	1.31
	$N_{y}$	2.00	2.43

## Appendix A

### Abbreviations and Symbols

$a_{1},\;a_{2},\;a_{4}$	Distance between centroid of the upper arm, forearm and the palm to its corresponding proximal joint (m)
$a_{3},\;a_{5}$	Position from the attached mass to the fixed joint of “absorber 1” and “absorber 2” (m)
$B_{beam},\;B_{0}$	Base of absorber’s cantilevered beam and the attached mass (m)
C	Damping coefficient matrix of the system (Nms/rd)
$C_{load},\;C_{reliab},\;C_{size},\;C_{surf},C_{temp}$	Load, reliability, size, surface and temperature correction factors
$c_{a},\;c_{1},\;c_{2},c_{3},\;c_{4}$	Bending damping coefficient of absorber’s beam and shoulder, elbow, biceps brachii and wrist muscles (Nms/rd)
$c_{a_{1x}},\;c_{a_{2x}}$	Linear damping coefficients of “absorber 1” and “absorber 2” (Ns/m)
DBS	Deep brain stimulation
DOF	Degree of freedom
DVA	Dynamic vibration absorber
$E_{beam}$	Modulus of elasticity of absorber’s beam material (GPa)
$F_{i},\;F_{c_{i}}$	Generalized conservative and frictional moments (Nm)
$F_{1},\;F_{2}$	Input moment at shoulder and wrist joints (Nm)
f	Vector of bending moment functions (Nm)
$H_{beam},\;H_{0}$	Height of absorber’s cantilevered beam and the attached mass (m)
H(ω)	Complex transfer function (rd/Nm)
$I_{beam}$	Area moment of inertia of the beam (m^4^)
$I_{1},\;I_{2},\;I_{4}$	Mass moment of inertia of the upper arm, forearm and the palm (kgm^2^/rd)
K	Stiffness coefficient matric of the system (Nm/rd)
$k_{a},\;k_{1},\;k_{2},k_{3},\;k_{4}$	Bending damping coefficient of absorber’s beam and shoulder, elbow, biceps brachii and wrist muscles (Nm/rd)
$k_{a_{1x}},\;k_{a_{2x}}$	Linear stiffness coefficients of “absorber 1” and “absorber 2” (N/m)
$k_{f}$	Fatigue stress concentration factor
$L_{beam},\;L_{0}$	Length of absorber’s cantilevered beam and the attached mass (m)
$l_{a},\;l_{3}$	Distance between absorber’s joint and controller device to the elbow joint (m)
$l_{1},\;l_{2},\;l_{4}$	Distance from the location of concentrated masses of the upper arm, forearm and the palm to its corresponding proximal joint (m)
M	Mass matrix of the system (kgm^2^/rd)
$m_{a_{1}},\;m_{a_{2}}$	Effective proof mass of “absorber 1” and “absorber 2” (kg)
$m_{0}$	Mass attached to absorber’s beam (kg)
$m_{1},\;m_{2},m_{3},\;m_{4}$	Masses of the upper arm, forearm, controller device and the palm (kg)
$M_{a}$	Total mass of the absorber (kg)
$N_{f},\;N_{y}$	Fatigue and yielding safety factors
PD	Parkinson’s disease
$q_{i},\;{\dot{q}}_{i}$	Generalized coordinates of angular displacement and velocity (rd) and (rd/s)
R	Raleigh dissipation function (J)
$S_{e},\;S_{e}^{\prime }$	Corrected and uncorrected endurance limit of the absorber’s beam (MPa)
$S_{ut},\;S_{y}$	Ultimate and yielding strength of absorber’s beam (MPa)
s	Proportional constant relating stiffness and damping coefficients
T, U	Kinetic and potential energy of the system (J)
${ℳ}_{a}$	Bending reaction moment on absorber’s beam (Nm)
$\alpha_{i}$	Receptance transfer function (rd/Nm)
$\theta ,\;\dot{\theta },\ddot{\theta }$	Angular displacement, velocity and acceleration functions (rd), (rd/s) and (rd/s^2^)
Θ	Angular displacement magnitude (rd)
$\Theta_{contreolled},\;\Theta_{uncontreolled}$	Angular displacement magnitude of the controlled and uncontrolled systems (rd)
ω	Driving frequency (rd/s)
$\omega_{a},\;\omega_{beam}$	Fundamental frequency of absorber’s system and its beam alone (rd/s)
$\omega_{m_{0}}$	Natural frequency of the attached mass (rd)
$\omega_{n}$	Natural frequency of the system (rd/s)
φ	Phase angle of the response (°)
σ	Bending stress (MPa)
ζ	Damping ratio
$\rho_{beam},\;\rho_{0}$	Density of the cantilevered beam of the absorber and the attached mass (kg/m^3^)
